# Electrocardiographic effects of class 1 selective histone deacetylase inhibitor romidepsin

**DOI:** 10.1002/cam4.467

**Published:** 2015-04-27

**Authors:** Philip T Sager, Barbara Balser, Julie Wolfson, Jean Nichols, Richard Pilot, Suzanne Jones, Howard A Burris

**Affiliations:** 1 Stanford University School of MedicineSan Francisco, California; 2 Veristat, LLCHolliston, Massachusetts; 3 Jean Nichols LLCSwampscott, Massachusetts; 4 Celgene CorporationSummit, New Jersey; 5 Sarah Cannon Research InstituteNashville, Tennessee

**Keywords:** Antiemetics, electrocardiography, electrolytes, T-cell, lymphoma, QTc, romidepsin

## Abstract

Romidepsin is a histone deacetylase inhibitor approved by the FDA for the treatment of patients with cutaneous or peripheral T-cell lymphoma who have received prior systemic therapy. The objective of this analysis was to evaluate the potential QTc effects of romidepsin. Patients with advanced malignancy received 4-h infusions of 14 mg/m^2^ romidepsin on days 1, 8, and 15 of a 28-day cycle. In cycle 2, a subset of patients received 1-h infusions of 8–12 mg/m^2^ romidepsin. Patients were administered antiemetics before each romidepsin dose and electrolyte supplementation as needed. Electrocardiogram readings were performed prior to antiemetic administration, prior to romidepsin administration, and at specified time points over the subsequent 24 h. Romidepsin exposure and heart rate were also assessed. In the electrocardiogram-evaluable population, 26 patients received romidepsin at 14 mg/m^2^ over 4 h. The maximum mean increases from the preantiemetic baseline for QTcF and heart rate were 10.1 msec (upper 90% CI, 14.5 msec) and 18.2 beats per minute, respectively. No patient in this study had an absolute QTcF value >450 msec and only one patient had an increase from the preantiemetic baseline of >60 msec. There was a mild reduction in the PR interval and no meaningful changes in the QRS interval. Despite the use of QT-prolonging antiemetics, treatment with romidepsin did not markedly prolong the QTc interval through 24 h. Increases in calculated QTc may have been exaggerated as a consequence of transient increases in heart rate.

## Introduction

Romidepsin is a potent, class 1 selective histone deacetylase (HDAC) inhibitor 1–3 approved by the US Food and Drug Administration (FDA) for the treatment of patients with cutaneous or peripheral T-cell lymphoma (CTCL, PTCL) who received ≥1 prior systemic therapy 4. Approvals were based primarily on pivotal phase 2 studies in each indication, which demonstrated durable single-agent activity 5,6.

Several HDAC inhibitors are under clinical development 1; pan-HDAC inhibitors vorinostat and belinostat 3 are also FDA approved in relapsed/refractory CTCL 7 and PTCL 8, respectively. Electrocardiogram (ECG) changes, including ST-segment and T-wave changes and corrected QT (QTc)-prolongations, have been described with various HDAC inhibitors 9–20 and a class effect has been suggested 9,10,18. However, few published systematic QTc studies have been performed with these agents, and some cardiac-related events that initially raised concern were recharacterized 10–12. Additionally, ECG analysis of the QT interval can be complex in older patients with significant baseline ECG ST-T wave abnormalities.

Prior to clinical development of romidepsin, in vitro electrophysiological assays were performed to assess the potential risk of QT prolongation. At 10 *μ*g/mL, romidepsin was shown to inhibit the hERG-related current by 37%. However, as this was ≈27-fold the maximum serum concentration (*C*_max_) in humans at the clinically administered dose (14 mg/m^2^ as a 4-h intravenous [IV] infusion 4) and romidepsin is 92% to 94% protein bound, this finding did not appear to be highly predictive of significant QTc prolongation. In guinea pig papillary muscle, action potential duration shortening was observed at 10 *μ*g/mL. Mild increases in heart rate were observed at all doses of romidepsin studied in dogs, and QTc intervals were increased in some dogs that received the highest dose tested, 1.0 mg/kg (20 mg/m^2^) (data on file, Celgene Corporation).

In a phase 1 trial, reversible T-wave flattening or inversions were observed and 1 patient had an asymptomatic 5-beat run of nonsustained ventricular tachycardia 21. In this trial, prophylactic antiemetics were necessary beginning with the 3.5 mg/m^2^ dose, the same dose at which QTc changes were first noted 21. It was not possible to determine whether these events were drug-related or reflected underlying characteristics of the patients 21. Early in clinical development, sudden death was reported in six patients across several studies, though each of the six patients had clinical comorbidities considered to be independent risk factors and thus a putative role of romidepsin was not clear 14,15,19,20,22. Subsequently, routine cardiac monitoring was incorporated into phase 2 studies and patients with significant cardiac disease were excluded 19. Protocols also required that potassium and magnesium be maintained in the high normal range, as hypokalemia and hypomagnesemia may be associated with ECG abnormalities 19–27. Following the implementation of these protocol modifications, no further sudden deaths, sustained ventricular tachycardia, or torsade de pointes were reported during clinical development.

As a postmarketing requirement, the FDA requested additional evaluation of the potential for romidepsin to prolong QT; that analysis is summarized in this report. This study was a substudy of trial GPI-06-0005, an open-label, phase 1 study evaluating the bioavailability of oral romidepsin and the pharmacokinetics (PK), tolerability, and safety of romidepsin. The objective of this analysis was to evaluate the potential QTc effects of romidepsin using a centralized laboratory ECG analysis. Because romidepsin is routinely administered after prophylactic antiemetics that can prolong the QTc 28,29, changes in QTc from both preantiemetic and postantiemetic/preromidepsin baselines were examined.

## Methods

### Study design

Adult patients with measureable or evaluable disease, a histologically confirmed diagnosis of advanced malignancy, and Eastern Cooperative Oncology Group performance status 0–2 were eligible. In cycle 1, all patients received 14 mg/m^2^ romidepsin as a 4-h IV infusion on days 1 (primary analysis data set), 8, and 15 of a 28-day cycle. In cycle 2, a subset of patients received romidepsin as a 1-h infusion, with the first cohort receiving 8 mg/m^2^ followed by escalation to 10 and 12 mg/m^2^ if the dose was well tolerated without dose-limiting toxicities. Most patients were given prophylactic antiemetics of the investigator’s choosing before each romidepsin dosing. Patients also received magnesium and potassium supplementation to ensure serum potassium was ≥3.8 mmol/L and magnesium was ≥2.0 mg/dL. The protocol, informed consent form, and other study documentation were reviewed and approved by the appropriate Institutional Review Board and the study was conducted in accordance with the Declaration of Helsinki. All patients provided written informed consent.

### Pharmacokinetic analysis

PK sampling was performed on day 1 of cycles 1 and 2, prior to and at 0.25, 0.5, 1, 2, 3, 4, 6, 8, and 24 h postinitiation of romidepsin administration. Plasma levels of romidepsin were assessed by Osaka Laboratory (JCL Bioassay Corporation, Osaka, Japan) using a validated liquid chromatography-tandem mass spectrometry assay. Romidepsin exposure was determined using standard noncompartmental methods as implemented in WinNonlin (v5.1, Pharsight Corporation, Mountain View, CA).

### Electrocardiograms

On day 1 cycle 1, triplicate ECG tracings (recorded 5–30 min apart) were obtained after ≥3 min of recumbency prior to antiemetic administration (preantiemetic baseline), within 1 h prior to romidepsin administration (postantiemetic/preromidepsin baseline), and at each PK sampling time postbaseline. On day 1 cycle 2, ECGs were not generally collected prior to romidepsin administration. PK/PD modeling is not reported as it was not possible to account for the possible effects of coadministered antiemetics and the absence of closely collected ECGs during times of PK sampling when rapid changes in the plasma concentrations occurred. For the central tendency and categorical analyses, the mean of the three replicates was used. All paper ECGs were analyzed by a core central ECG laboratory (Duke Clinical Research Institute [DCRI], Durham, NC) in a fully blinded fashion. A DCRI technician marked the fiducial points on each ECG tracing and, using a digital caliper, measured the QT, PR, RR, and QRS intervals. All ECGs were over read by a DCRI cardiologist for verification of the interval measurements.

### Statistical analyses

Standard analyses were performed as defined in the International Conference on Harmonization E14 Guidance (ICH-E14) 30. The central tendency analysis examined the changes from preantiemetic and postantiemetic/preromidepsin baselines, and QTc categorical analysis examined the number of participants reaching a QTc increase compared with baseline or absolute thresholds of QTcF. QTcF was prospectively used as the methodology to correct the QT interval for changes in heart rate. For PK variables, the exposure metric employed was the patient-, regimen-, and time-specific observed plasma concentration of romidepsin. The PK-evaluable population included all patients who received ≥1 dose of romidepsin and had ≥1 postdose PK observation. The ECG-evaluable population included all patients who received ≥1 dose of romidepsin and had ≥1 baseline ECG on cycle 1 day 1, and ≥1 postdose, centrally read ECG assessment. For the central tendency and outlier analyses, statistical evaluations were performed using SAS v9.1 or higher (SAS Institute Inc, Cary, NC) and continuous ECG data were summarized using descriptive statistics. A 90% CI for ΔQTcF was considered the primary end point; the upper bound at each time point was compared with a 20-msec threshold, which is often used for oncologic compounds where QTc increases >10 and <20 msec have been considered to have a positive benefit:risk ratio 31. Changes in PR and QRS intervals and heart rate were also measured. The impact of heart rate on QT was examined by a linear regression of QTcF versus RR intervals.

## Results

### Patient characteristics

Twenty-nine patients with advanced malignancies (bladder, breast, lung, ovarian, prostate, or other carcinoma; B-cell lymphoma; sarcoma) received romidepsin at 14 mg/m^2^ as 4-h infusions and were eligible for PK analysis. The first three patients enrolled in the study did not have centrally read ECG assessments, thus 26 patients were ECG- and exposure-response-evaluable. During cycle 1, 284 sets of ECGs were collected, with triplicate ECGs at 97% of the time points. Both the mean and median times between the first and third ECG measurements were 11.3 min (95% CI, 10.9–11.7 min).

During cycle 2, 14 patients received romidepsin at doses of 8–12 mg/m^2^ as 1-h infusions: 3 at 8 mg/m^2^, 6 at 10 mg/m^2^, and 5 at 12 mg/m^2^. Data from 1 patient given 8 mg/m^2^ were excluded from the ECG analysis because ECGs were obtained at significant deviations from the protocol-specified time points. Additionally, ECGs at the preantiemetic baseline were only performed in 2 of 14 patients prior to 1-h infusions. Thus, the cycle 1 data set formed the primary ECG analysis.

Baseline characteristics for ECG-evaluable patients are presented in Table1. The majority of patients were female (62%) and white (89%), and their ages ranged from 44 to 82 years. Twenty of the 26 patients (77%) received antiemetic premedication on cycle 1 day 1 (as well as during other cycles), including 5-HT3 antagonists (24 mg ondansetron, 17 [65%]; 1 mg granisetron, 1 [4%]; 24 mg ondansetron + 1 mg granisetron, 1 [4%]); and dopamine antagonists (10 mg prochlorperazine, 1 (4%]). Eighteen of the 26 patients (69%) had a history of or ongoing cardiovascular abnormalities.

**Table 1:** Baseline demographics, ECG-evaluable population

Characteristic	4-h Infusion, 14 mg/m^2^ (*n* = 26)	1-h Infusion		
		8 mg/m^2^ (*n* = 3)	10 mg/m^2^ (*n* = 6)	12 mg/m^2^ (*n* = 5)
Sex, *n* (%)				
Male	10 (38)	0	2 (33)	3 (60)
Female	16 (62)	3 (100)	4 (67)	2 (40)
Age in years, median (range)	60 (44–82)	52 (45–77)	65 (50–76)	68 (46–82)
Race, *n* (%)				
White	23 (88)	2 (67)	5 (83)	5 (100)
Black	3 (12)	1 (33)	1 (17)	0

### Romidepsin pharmacokinetics

**Figure 1:**
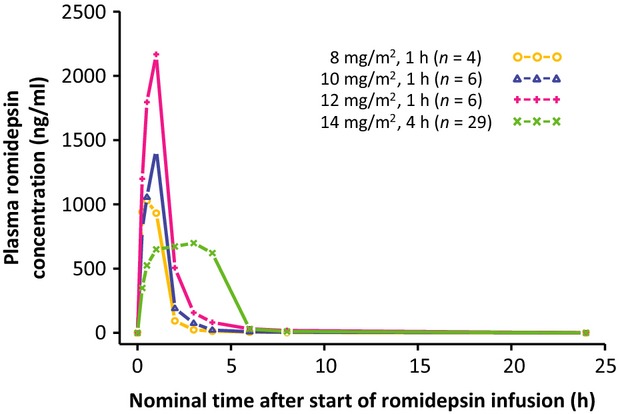
Median plasma romidepsin concentration versus time.

Exposure to romidepsin following 4-h or 1-h infusions is shown in Figure1 and Table2. The median *C*_max_ for 1-h infusions of 8, 10, and 12 mg/m^2^ were 1.4-fold, 1.9-fold, and 2.7-fold higher, respectively, than the median *C*_max_ for 4-h infusions of 14 mg/m^2^. Additionally, the median *C*_max_ for 1-h infusions of 10 mg/m^2^ or 12 mg/m^2^ were higher than the highest *C*_max_ reported with 4-h infusions of 14 mg/m^2^.

**Table 2:** Romidepsin pharmacokinetic parameters by dose regimen

Parameter	4-h Infusion, 14 mg/m^2^	1-h Infusion		
		8 mg/m^2^	10 mg/m^2^	12 mg/m^2^
Median *C*_max_ (range), ng/mL	779.5 (393.9–1335)	1107 (1011–1193)	1480 (592.6–1797)	2094 (948.9–2668)
Median AUC_0-∞_ (range), h × g/mL	3066 (1605–5670)	1352 (1299–1805)	1779 (686.0–2439)	3012 (1178–5068)
Median *t*_1/2*λ*z_ (range), h	3.70 (2.92–4.22)	4.82 (4.34–5.18)	4.94 (4.24–5.54)	4.32 (3.96–4.77)

AUC_0-∞_, area under the plasma concentration versus time curve; *C*_max_, maximum plasma concentration; *t*_1/2*λ*z_, apparent terminal half-life.

### Heart rate

**Figure 2:**
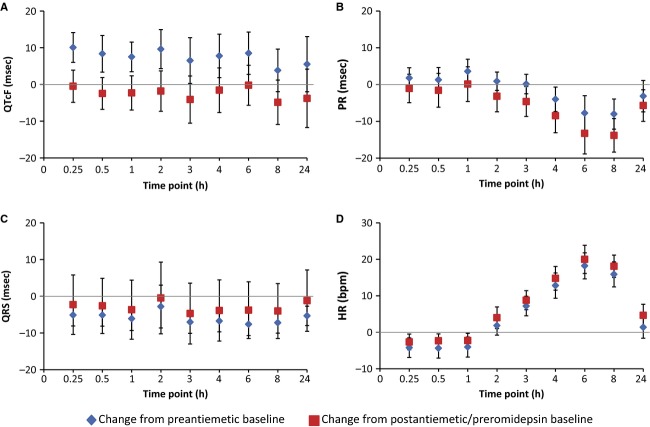
Mean change (90% CI) in (A) QTcF, (B) PR, (C) QRS, and (D) heart rate from baseline over time following dosing of romidepsin 14 mg/m^2^ IV over 4 h, ECG-evaluable population.

With 4-h 14 mg/m^2^ romidepsin dosing, the mean heart rate began to increase at the mid-point of the infusion (2-h time point; Table3, Fig.2). The maximum mean increase in heart rate from both the preantiemetic and postantiemetic/preromidepsin baselines occurred at the 6 h time point, at 18.2 and 20.0 beats per minute (bpm), respectively. At the 8 h time point, the heart rate change from baseline remained near maximum, but at 24 h, the mean increase in heart rate had dropped to near baseline. Results obtained following the 8, 10, and 12 mg/m^2^ 1-h dosing regimens were similar, with a maximum mean increase in heart rate from the postantiemetic/preromidepsin baseline of 19.2, 16.5, and 18.2 bpm, respectively, at the 6 h time point.

**Table 3:** QTcF, PR, QRS, and heart rate over time following dosing of romidepsin 14 mg/m^2^ IV over 4 h, ECG-evaluable population

ECG time point	*n*	QTcF (msec), mean (SD)	PR (msec), mean (SD)	QRS (msec), mean (SD)	HR (bpm), mean (SD)
Preantiemetic	23	390.9 (26.5)	150.5 (31.6)	81.3 (24.1)	82.0 (15.0)
Postantiemetic/preromidepsin	24	400.6 (23.4)	155.7 (33.4)	78.4 (14.2)	78.8 (14.1)
0.25 h	25	399.8 (21.8)	154.7 (32.9)	74.8 (15.5)	77.7 (13.8)
0.5 h	26	398.1 (21.2)	153.5 (31.7)	74.7 (15.5)	77.9 (14.3)
1 h	26	397.8 (20.1)	155.8 (33.6)	73.7 (13.4)	77.6 (13.8)
2 h	25	397.7 (21.0)	154.4 (31.2)	74.9 (18.9)	84.6 (14.7)
3 h	25	394.9 (20.0)	153.0 (30.9)	71.1 (11.3)	89.6 (14.6)
4 h	24	397.8 (21.4)	149.8 (30.8)	72.2 (10.0)	95.2 (15.4)
6 h	26	399.8 (22.7)	141.9 (28.0)	73.4 (10.6)	100.5 (16.2)
8 h	26	394.8 (20.5)	142.0 (29.4)	73.3 (13.4)	98.1 (14.0)
24 h	25	398.5 (22.8)	149.6 (32.1)	75.4 (12.4)	83.0 (14.6)

bpm, beats per minute; ECG, electrocardiogram; IV, intravenous; QTcF, QT interval corrected for heart rate using Fridericia’s formula; SD, standard deviation.

### QTc, PR, and QRS analysis of central tendencies

#### Cycle 1

The QTc central tendency analysis (Table3, Fig.2) of data from patients given 14 mg/m^2^ romidepsin as a 4-h infusion showed a 9.7-msec increase between the mean preantiemetic (*n* = 23) and the postantiemetic/preromidepsin (*n* = 24) QTcF baselines. Thus, given the confounding effect of antiemetics on the QTc interval and their rapidly falling plasma concentrations following administration, the conservative and most appropriate approach to analysis is to use the preantiemetic measurements as the baseline. The maximal mean QTcF increase from the preantiemetic baseline was 10.1 msec (90% upper CI, 14.5 msec) at 0.25 h postinitiation of romidepsin, excluding an effect >20 msec. The mean changes from the postantiemetic/preromidepsin baselines were smaller. As expected, the QTcF demonstrated some dependency on heart rate, particularly at more rapid heart rates. A plot of QTcF interval versus RR interval had a linear regression slope of 0.06 and Y intercept of 347 msec. We observed small reductions in the PR interval associated with heart rate increases and no meaningful changes in the QRS intervals following romidepsin administration at 14 mg/m^2^ as a 4-h infusion (Table3, Fig.2).

#### Cycle 2

For patients receiving romidepsin as a 1-h infusion, no trends were observed across dose levels for mean changes in QTcF from postantiemetic/preromidepsin baseline. Maximum mean changes were 7.6 msec at 4 h, 3.1 msec at 2 h, and 4.5 msec at 4 h for 8, 10, or 12 mg/m^2^, respectively. Changes in QTcF from preantiemetic baseline could not be assessed as only 2 of the 14 patients who received 1-h dosing had preantiemetic ECG readings.

### ECG categorical analysis

The categorical analysis demonstrated that no patient who received 4-h or 1-h romidepsin infusions at any dose had an absolute QTcF value >450 msec (Table4). Four of the 26 patients who received 14 mg/m^2^ as a 4-h infusion had increases from the preantiemetic baseline in QTcF ≥30 msec, including one patient with an increase >60 msec.

**Table 4:** Categorical analysis of maximum change in QTcF from baseline following dosing of romidepsin

Maximum change	Cycle 1 day 1 14 mg/m^2^ IV over 4 h (*n* = 26)	Cycle 2 day 1 8, 10, and 12 mg/m^2^ IV over 1 h (*n* = 14)^1^
QTcF change from preantiemetic baseline, *n* (%)		
<30-msec increase	17 (65.4)	NA
30–60-msec increase	3 (11.5)	NA
>60-msec increase	1 (3.8)	NA
Missing^2^	5 (19.2)	NA
QTcF change from postantiemetic, preromidepsin baseline, *n* (%)		
<30-msec increase	23 (88.5)	13 (92.9)
30–60-msec increase	1 (3.8)	1 (7.1)
>60-msec increase	0	0
Missing^2^	2 (7.7)	0
QTcF absolute value, *n*		
>450 msec	0	0

NA, not assessed; QTcF, QT interval corrected for heart rate using Fridericia’s formula.

1 Only 2 of 14 patients who received romidepsin as a 1-h infusion had preantiemetic baseline.

2 Did not have postbaseline data available for assessment.

## Discussion

In this analysis, the potential of romidepsin to elicit QTc changes was studied via examination of the central tendency of QTc, PR, or QRS and changes in heart rate over time and a categorical analysis of QTc relative to standard thresholds. The primary analyses focused on 4-h dosing at 14 mg/m^2^ as this is the currently approved dose 4, both preantiemetic and postantiemetic/preromidepsin ECG data were available, and there were more evaluable patients. Data for 1-h dosing are secondary and support the primary analysis.

For patients who received 4-h 14 mg/m^2^ romidepsin IV dosing, the QTc central tendency analysis demonstrated a 9.7-msec mean increase between preantiemetic and postantiemetic/preromidepsin baselines, consistent with the well-known effects of certain antiemetics (including ondansetron) on the QTc interval 28,29. The majority of patients (18/26) received ondansetron 24 mg IV. Published QT results for ondansetron 32 mg IV demonstrated a marked initial increase (≈20 msec) in QTc that rapidly declines and was ≈6 msec at 4 h 32. Thus, 24 mg ondansetron likely results in a QTc effect of <5 msec at 4 h. The plasma concentration of romidepsin with 4-h 14 mg/m^2^ IV dosing rapidly increased, remained relatively stable until the end of the 4-h infusion, and then fell rapidly (Fig.1). Thus, the 4-h time point (mean increase of 7.76 msec from preantiemetic baseline) may more accurately reflect the impact of 4-h IV romidepsin dosing on the QTc interval.

According to ICH-E14, the threshold for regulatory concern for increased QTc is upper bound of the 90% CI for the change from baseline (placebo adjusted) of >10 msec 30, which correlates with negligible risk of drug-induced proarrhythmia. However, this threshold is not appropriate for benefit:risk assessment of oncology agents which may provide life-saving benefits. Thus, a 20-msec threshold for meaningful clinical relevance has been commonly used for patients receiving nonadjuvant oncology agents 31. Despite the use of QT-prolonging antiemetics, the QTc interval following 4-h 14 mg/m^2^ romidepsin IV dosing was only moderately increased (maximum mean increase of 10.1 msec; upper bound of the 90% CI, 14.5 msec) compared with the preantiemetic baseline, and below the 20-msec threshold. Using the preantiemetic baseline is the most conservative and clinically relevant approach, even though it likely results in exaggeration of the actual QTc effect of romidepsin. Whereas sophisticated PK/PD modeling could potentially adjust for the antiemetic effects, this was not possible (see Methods) 33. The categorical QTc analysis showed no patient with a QTcF >450 msec and one patient with an increase of >60 msec from the preantiemetic baseline. Although the patient numbers are small, administration of romidepsin at 8–12 mg/m^2^ with 1-h dosing permitted evaluation of QTc at supratherapeutic romidepsin concentrations and did not show an exaggerated response compared with therapeutic dosing on cycle 1 day 1.

Romidepsin treatment was also shown to moderately increase heart rate (up to ≈20 bpm), particularly at the 3 through 8 h time points, as well as in other studies 19,20,23. The reasons for the apparent delay in response are not clear and may be a direct elecrophysiologic effect, the effect of a metabolite, or perhaps related to adverse events (e.g., nausea/vomiting). The Fridericia method for correcting the QT interval for heart rate is often not fully adequate in the setting of substantial heart rate increases and commonly results in overcorrection and inflated QTcF increases, independent of repolarization 34.

In contrast to the ICH-E14 stipulated methodology, this study was not placebo-controlled or double-blinded, nor did it use an active control for the assessment of assay sensitivity 30,35. Although such approaches would be ideal, they are not suitable for antineoplastic agents (including romidepsin) due to safety and ethical concerns. Thus, in oncology, open-label designs that compare on-drug QTc to predrug baseline are common 36–38. ECGs were analyzed by a core ECG laboratory in a fully blinded fashion to remove any potential for bias. However, this study does have limitations. The patient number is relatively small, antiemetic medications were not administered using a fixed protocol, antiemetic levels were not measured, and the triplicate ECGs were performed too far apart to permit exposure-response modeling. There is a need in future oncology studies using antiemetic medications to prospectively incorporate exposure-response methodologies that separate the QTc effects of concomitant medications from those of the investigational agent and to employ sophisticated approaches that correct the QT interval in the setting of significant heart rate increases, given the potential inadequacy of fixed correction approaches in this situation 34,39,40. A strength of our study is that it mimics real-life romidepsin administration: with QTc-prolonging antiemetics to prevent nausea, as is the norm for many chemotherapeutic agents. Thus, the central tendency results on day 1 cycle 1, which included 18 patients with cardiac disease, are representative of the QTc effect of romidepsin in clinical use.

Other published reports have shown that treatment with romidepsin was not associated with myocardial damage or impaired cardiac function, although romidepsin has been previously associated with mild QTc effects, possibly exaggerated by increased heart rate and concomitant antiemetic administration 19,20,23. One analysis of patients with PTCL (*n* = 84) and CTCL (*n* = 47) reported frequent ST-T wave changes, possibly due in part to electrolyte reductions 20. This study highlighted the need to consistently monitor and supplement electrolytes as necessary when using romidepsin in this patient population (per protocol, 92% of the patients required electrolyte supplementation at some time during this study to maintain levels in the normal range) 20.

In conclusion, despite previous concerns regarding prolonged QTc with HDAC inhibitors 9,10,18, this study suggests that with romidepsin treatment at the clinically administered regimen, QTc was not markedly prolonged and may have been exaggerated by transient increases in heart rate and concomitant antiemetic administration. The potential of romidepsin to mildly prolong the QTcF should be considered if QTc-prolonging antiemetics are being used or higher plasma exposures are anticipated (as can occur when strong CYP3A4 or P-glycoprotein inhibitors are coadministered). Additionally, potassium and magnesium levels should be maintained within the normal range. Romidepsin is associated with transient moderate heart rate increases, which should be taken into account during patient management.
